# The associations between social support change and physical activity trajectory from late adolescence to young adulthood

**DOI:** 10.1186/s12889-023-16422-z

**Published:** 2023-08-07

**Authors:** Juan Cao, Kun Wang, YuHui Shi, YuQing Pan, MoHan Lyu, Ying Ji

**Affiliations:** 1 Department of Social Medicine and Health Education, School of Public Health, Peking University, Beijing, 100191 China; 2 Department of Disease Control and Prevention, Peking University Third Hospital, Beijing, 100191 China; 3 Faculty of Science, The University of Sydney, Sydney, 2006 Australia

**Keywords:** Physical activity trajectory, Family support change, Peer support change

## Abstract

**Background:**

Previous research examined the associations between social support and physical activity. However, little is known about the associations between social support change and trajectories of physical activity during the transition from late adolescence to young adulthood.

**Methods:**

The current study sought to examine these issues among 434 Chinese college students (*M*_age_ = 19.15, *SD*_age_ = 0.61; 46.1% male), who completed questionnaires regarding demographics, physical activity, family support change, and peer support change across three waves (the data from one of the waves was retrospective).

**Results:**

After controlling for covariates, the findings revealed that: (a) there was an increase in overall physical activity and duration, but a decrease in frequency during the transition from late adolescence (the second year of high school) to young adulthood (the third year of college); (b) family support change did not contribute to trajectories of physical activity, while peer support change significantly predicted the trajectory of overall physical activity, duration, and frequency.

**Conclusions:**

The findings extend the literature on physical activity from a developmental perspective by revealing different trends among physical activity duration and frequency, and unpacking different effects of family and peer support change on trajectories of physical activity.

**Supplementary Information:**

The online version contains supplementary material available at 10.1186/s12889-023-16422-z.

## Background

Regular physical activity, as an important health-promoting behavior, prevents chronic illnesses and obesity, reduces depressive and anxiety symptoms, and improves subjective well-being and life satisfaction [1–3]. Despite the benefits of physical activity, it changes dynamically across the lifespan [4]. Evidence from longitudinal studies suggested that there is a decline in the duration and frequency of physical activity in late adolescence through young adulthood, compared to other age ranges [5, 6]. Previous research has examined the predictors of physical activity during adolescence or young adulthood [7–9]. However, few studies have explored the contributors to the change in physical activity over time (i.e., physical activity trajectory), especially for the transition from late adolescence to young adulthood. The transition period is an important time to develop unhealthy habits (e.g., inactive physical activity) as a result of psychosocial changes, including personal identity formation, changing relationships with parents and peers, and academic and employment transitions [10, 11].

Among all the social and environmental factors affecting physical activity

[12], social support is one of the most prominent factors for maintaining and/or initiating physical activity change [13]. Higher levels of social support can lead to increased opportunities for social interaction, positively influence health-behavior decision-making, and increase access to resources related to physical activity, thereby promoting physical activity. Although most empirical studies have supported this idea [14–16], several considerations need further research. Most of the prior studies regarded social support as a static construct [15], and there is a dearth of evidence on how the change in social support shaped physical activity trajectory over time. The dynamic properties of social support (i.e., decay, growth, and staticity) involve different underlying social processes and thus have different effects on individuals’ developmental outcomes (e.g., physical activity) [17]. Given that family and peers are the main sources of supporting physical activity among adolescents and early adults [18, 19], it is necessary to simultaneously explore the influence of family and peer support change on the trajectory of physical activity, which is addressed in the current study.

Further, WHO (World Health Organization) guidelines recommend ≥ 150 min/per week of moderate to vigorous physical activity for adults (including early adults), which is the resultant of the frequency (≥ 5/week) × the duration (≥ 30/each bout) [20]. However, most of the prior studies assessed overall physical activity by combining all components (e.g., duration, frequency) into a single statement, which is difficult to disentangle the differential determinants of distinct components of physical activity and interventions cannot be structured accordingly [6]. Empirical research indicated that both the duration and frequency of bouts of physical activity have important implications for physical and mental health (e.g., lower risk of depression, higher life quality) [21, 22]. However, there is an optimal pattern in frequency and duration of physical activity, with greater benefits if physical activity periods are either long or frequent [23]. For example, some studies observed that with duration and intensity constant, increasing frequency by one time per week was significantly associated with decreased total cholesterol and BMI [24], other studies indicated that accumulating physical activity in bouts may confer health benefits beyond that of physical activity accumulated more sporadically [25]. These principles suggest that the health benefits of physical activity depend on the individual’s levels of duration and frequency of physical activity. Further, differences in the frequency and duration of physical activity may help to explain differences in overall physical activity due to their different determinations [26]. For example, self-efficacy could facilitate frequency (not duration) and intention only explained duration [27], and the physical activity intervention increased the odds of the frequency of physical activity but not the duration during the intervention period and the follow-up period [28]. Therefore, it seems important to evaluate the changes in frequency and duration of bouts of physical activity and their associations with social support changes, which is essential for better understanding intervention effects and the development of physical activity recommendations.

In light of previous research on the associations between social support and physical activity, the aims of this longitudinal study were: 1) to explore the trajectories of physical activity (i.e., overall physical activity, duration, and frequency) during the transition from late adolescence (e.g., high school) to young adulthood (e.g., college); and 2) to examine how social support (i.e., family support and peer support) changes are associated with the trajectory of physical activity.

## Method

### Participants

Chinese college students were recruited from a public university in Beijing, China, with more than 16,000 undergraduates. A stratified cluster sampling method was adopted to acquire subjects. The sampling was taken under the stratification of academic major because of previous research showing differences in physical activity by major [29], and three academic majors (i.e., social sciences, science and engineering, and medicine) were selected. Further, cluster sampling was conducted by classes of these majors. To balance the sample size of the three academic majors, a minimum sample size of 150 college students was randomly selected from each sample major. At Time 1 (T1, spring 2019, the first year of college), the self-administered electronic web-based questionnaires were sent via the WeChat platform (a smartphone application; Tencent Holdings Ltd., China) to 468 college students from 14 classrooms of three academic majors above, with the number of students ranging from 7 to 89 in each classroom. Nine subjects did not respond despite several reminders, and a total of 459 students participated in the study at Time 1 and provided retrospective information about their physical activity from two years before Time 1 (Time 0, T0, spring 2017, the second year of high school). Eight subjects were excluded from the 459 initially participating in the survey since they were not the interested subjects (e.g., not a freshman), and 451 subjects (freshman; 46.8% male; *M*_age_ = 19.17, *SD*_age_ = 0.63) remained. Of the T1 participants, 434 (3.77% attrition rate) participated in the study at Time 2 (T2, spring 2021, the third year of college). Attrition was mainly due to withdrawal, joining the army, or dropping out according to students’ teachers. Comparisons among participants who participated in T1 assessment and those who participated in both T1 and T2 assessment indicated no significant differences in all studied variables and demographic variables (*p*s > 0.05). These results suggest that the missingness was random or was potentially a result of other unmeasured variables. Therefore, a subsample of 434 students (46.1% male) was analyzed in the present study.

### Procedures

The study followed the Ethics Committees’ guidelines and was approved by the university Institutional Review Board (No. IRB00001052-19019). Informed consent from participants was gathered before survey data collection. Data were collected through self-administered electronic web-based questionnaires, which were issued utilizing the WeChat platform. Participants were informed to withdraw from the study at any time if they do not wish to participate.

## Measures

### Demographic variables

Gender (0 = male, 1 = female), parental education level (maximum value for mother or father; ranging from 1 = middle school and below to 4 = college and above), family monthly income, and body mass index (BMI; retrospective and self-reported) in participants’ second year of high school (Time 0) were all considered when examining the effects of parent and peer support change on physical activity trajectory, given their impacts on physical activity of college students [30, 31].

### Physical activity (Time 0, Time 1, Time 2)

In the study, physical activity was defined as self-disciplined, planned, and repetitive physical activity with a certain intensity, frequency, and duration. Physical activity was measured using the Chinese revised version of the Physical Activity Rating Scale [32]. Participants provided retrospective information about their physical activity from two years before Time 1 (Time 0; spring 2017, the second year of high school), during the past one month at Time 1 (spring 2019, the first year of college), and during the past one month at Time 2 (spring 2021, the third year of college). Although the assessment of physical activity in the second year of high school was retrospective, a previous study indicated that young adults can recall physical activity of several years ago with good reliability, such that the correlation coefficients between physical activity of 2–3 years ago and current physical activity was high (ranged 0.64 to 0.84) [33], and the proportion who recalled the same activity level as originally reported ranged from 69 to 96% [34]. Further, distantly recalled physical activity was often used in previous studies examining trajectories of physical activity [35, 36]. Items included: 1) Intensity: did you conduct light (e.g., walking, doing radio exercises)/low (e.g., jogging, doing Tai Chi)/moderate (e.g., running, play ping pong)/vigorous (e.g., badminton, basketball, tennis, running, aerobics) activities; 2) Duration: if yes, participants were asked to rate how much time you spent on physical activity each time on a 4-point scale, including 1 (≤ 10 min), 2 (11-30 min), 3 (31-60 min), and 4 (≥ 60 min), and 3) Frequency: participants was asked to rate how often you spent on physical activity on a 6-point scale, including 1(one time/month), 2 (2–3 times/month), 3 (one time/week), 4 (2–3 times/week), 5 (4–5 times/week), and 6 (almost once a day). The present study used the sum of the duration and frequency of four different-intensity physical activities to represent the duration and frequency, respectively. The overall score of physical activity = $\sum intensity\times duration\times frequency$. Possible scores ranged from 0 to 240 with higher scores reflecting more physical activity. Previous studies showed that the scale demonstrated adequate reliability (test–retest reliability was 0.82) in the Chinese population [37]. In the current study, the 2-year test–retest reliability for overall physical activity, duration, and frequency were 0.67, 0.80, and 0.76, respectively.

### Physical activity social support change ( Time 1)

Previous studies argued that using subjective social support was a common approach, in which social support can be conceptualized as a subjective perception of an individual’s support that they receive from others [38]. Thus, two types of subjective physical activity social support change were examined in the study: family support change and peer support change. Family support change was assessed by asking participants to respond to one question “How much support (encouragement, reminders, company, praise, equipment purchases, etc.) did your family provide for your physical activity 2 years ago?”. And peer support change was assessed by asking participants to respond to one question “How much support (encouragement, reminders, company, praise, equipment purchases, etc.) did your peer provide for your physical activity 2 years ago?”. Items were rated on a five-point Likert scale ranging from 1 (much higher than it is now) to 5 (much lower than it is now) with higher scores reflecting a greater increase in family and peer support over time. In the current study, a clear definition and concrete examples of social support were provided when subjects responded to the measures of social support change. Previous studies indicated that when a construct is unambiguous in scope, the use of a single item can be appropriate and “should not necessarily be considered unsound” [39]. Further, single-item measures have been used to assess social support in prior studies focusing on adults between the ages of 18 and 65 years (including college students) [40, 41].

### Data analytic strategy

A series of models were estimated using M*plus* Version 7.1 in the following sequence [42].

### Unconditional latent growth model

To examine the trajectories of physical activity (i.e., overall physical activity, duration, and frequency) across the second year of high school, first-year college, and third-year college, three unconditional latent growth curve models were separately conducted to determine whether average scores for overall physical activity, duration, and frequency change over time (i.e., increase, decrease, or remain constant). The trajectory of overall physical activity/duration/frequency was defined by the intercept (i.e., the average initial level of the target variables) and the slope (i.e., the average rate of change over time in the target variables) [43]. Three model fit indices were used: the Comparative Fit Index (CFI), the Root Mean Square Error of Approximation (RMSEA), and the Standardized Root Mean Square Residual (SRMR). CFI values greater than 0.90, and RMSEA / SRMR values less than 0.08 indicate an acceptable model fit [44, 45].

### Conditional latent growth model

**Fig. 1:**
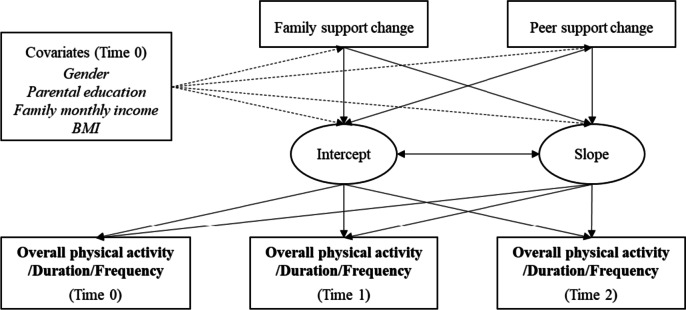
Conditional latent growth model for physical activity

Family support change, peer support change, and covariates were entered into the latent growth models to evaluate whether family and peer support change explained the intercepts and slopes for physical activity (i.e., overall physical activity, duration, and frequency) trajectories. A total number of three conditional latent growth models were run. Each model included all growth parameters (i.e., intercepts and slopes for physical activity) as dependent variables, sociodemographic covariates (i.e., gender, parental education level, family monthly income, and BMI) as control variables, family and peer support change as independent variables (see Fig. 1). The Bonferroni method was applied to control for multiple comparisons based on the number of independent tests.

## Results

### Descriptive analyses of samples physical activity

The final analytical sample included 434 students (46.1% male; Mean baseline age = 19.15 ± 0.61 years), who responded to measures of physical activity at T0, T1, and T2. Most of the participants (63.8%) had normal body mass index (i.e., 18.5 ~ 23.9). More than 50% of the participants reported that their parents had college and above, and family income is above 9000 RMB per month. According to the National Bureau of Statistics of China, the mean monthly family income was 9414 RMB for a family in Beijing in 2017 (i.e., the participants’ second year of high school). As such, most participants in the present study were from families with monthly incomes above the average city level. The descriptive information of the sample and physical activity was presented in Table 1.

**Table 1:** Descriptive analyses of samples and physical activity (*N* = 434)

	Frequency	Percent (%)	Physical activity (T0)			Physical activity (T1)				Physical activity (T2)			
			*M*	*SD*		*M*		*SD*		*M*			*SD*
*Gender*													
Female	234	53.9	28.74	25.95		31.76		27.41		34.33			26.57
Male	200	46.1	37.89	27.05		39.21		26.13		39.14		28.41	
*Parental education*													
Middle school and below	66	15.2	31.12	26.91		34.08		27.26		33.29			26.68
High school	75	17.3	23.26	23.26		30.40		24.49		27.45			25.25
Associated degree	66	15.2	24.39	24.39		36.97		27.58		34.20			26.18
College and above	227	52.3	28.26	28.26		36.59		27.61		41.18			28.02
*Family monthly income (T0)*													
Below 6000 RMB	116	27.2	27.42		20.51		31.78		26.53		31.06		25.00
6001 ~ 9000RMB	66	15.4	30.98		26.76		38.24		27.44		32.45		29.37
9001 ~ 12000RMB	66	15.5	30.30		23.34		31.68		26.00		38.48		27.56
12001 ~ 20000RMB	98	22.9	40.42		31.79		36.77		25.46		42.46		26.40
Above 20000RMB	81	17.4	35.44		29.61		37.72		28.33		39.89		29.72
*BMI (kg.m*^*−2*^*) (T0)*													
< 18.5	83	19.1	28.54		25.11		24.90		21.28		32.17		26.10
18.5 ~ 23.9	277	63.8	34.80		28.35		37.76		28.14		37.05		27.70
≥ 24	74	17.1	31.03		21.93		37.14		26.26		39.57		28.10
*Family support change (T0 → T1)*													
1 (much higher than it is now)	7	1.6	40.29		45.89		43.14		34.77		17.14		17.81
2 (a little higher than it is now)	28	6.5	43.29		25.23		33.96		28.90		41.57		30.03
3 (almost similar)	310	71.4	34.31		27.17		34.34		27.12		36.37		27.93
4 (a little lower than it is now)	78	18.0	26.24		22.31		38.10		24.41		37.77		25.08
5 (much lower than it is now)	11	2.5	11.45		16.45		36.82		35.28		32.27		28.44
*Peer support change (T0 → T1)*													
1 (much higher than it is now)	15	3.5	58.67		41.67		38.73		32.33		49.13		27.78
2 (a little higher than it is now)	72	16.6	40.39		23.72		31.19		23.44		33.74		24.75
3 (almost similar)	243	56.0	33.70		26.98		35.27		27.35		36.23		28.96
4 (a little lower than it is now)	80	18.4	22.38		20.82		37.35		25.77		36.50		25.96
5 (much lower than it is now)	24	5.5	22.42		22.07		37.04		34.85		40.42		24.39
Total			32.96		26.82		35.19		27.05		36.55		27.50

T0, T1, T2 = Time 0 (the second year of high school), Time 1 (the first year of college), and Time 2 (the third year of college), respectively. And 7 participants did not report their family monthly income

### Trajectories of physical activity

The study developed growth models to capture trajectories of physical activity over time. Results from the unconditional growth model for overall physical activity specifying intercept and linear slope demonstrated a good model fit (CFI = 1.00, SRMR = 0.01). In the model, the mean of the intercept was statistically significant (*b* = 33.02, *SE* = 1.24, *p* < 0.001), and the mean of the slope was also statistically significant (*b* = 1.77, *SE* = 0.76, *p* = 0.020), indicating that Chinese youth, on average, experienced an increase in engaging in physical activity during the transition from late adolescence to young adulthood.

**Fig. 2:**
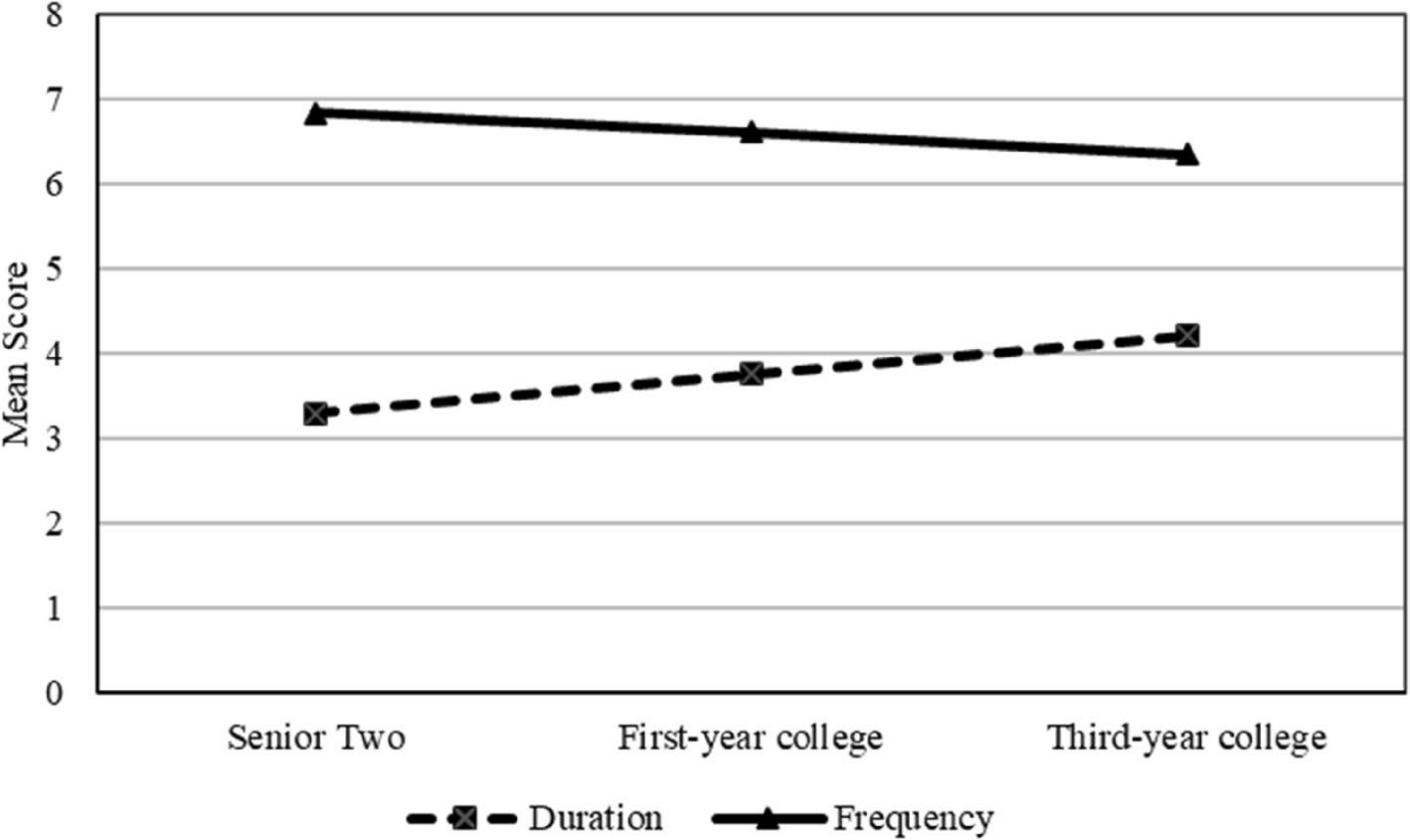
Unconditional growth model for physical activity frequency and duration

Given that physical activity is assessed based on multiple indicators (including duration and frequency), the present study further examined the trajectories of physical activity duration and frequency. Results from the unconditional growth models for duration showed that Chinese youth experienced an increase in the duration of individual bouts of physical activity as they make the transition from late adolescence to young adulthood (*b*_intercept_ = 3.30, *SE* = 0.11, *p* < 0.001; *b*_slope_ = 0.46, *SE* = 0.07, *p* < 0.001; CFI = 0.92, SRMR = 0.05). Figure 2 showed the modeled trajectory of physical activity duration over time, together with the mean duration of physical activity at each age. In contrast, they experienced a decline in the frequency of physical activity as they make the transition from late adolescence to young adulthood (*b*_intercept_ = 6.87, *SE* = 0.20, *p* < 0.001; *b*_slope_ = -0.26, *SE* = 0.12, *p* = 0.033; CFI = 0.98, SRMR = 0.02). Figure 2 showed the modeled trajectory of physical activity frequency over time, together with the mean frequency of physical activity at each age.

### Effects of social support change on trajectories of physical activity

Table 2 displays the model results examining the effects of social support change (i.e., family support change, peer support change) on trajectories of physical activity, along with controls for sociodemographic covariates, including gender, parental education level, family monthly income, and BMI.

**Table 2:** Effects of family support change and peer support change on trajectories of physical activity

	Overall physical activity					Duration				Frequency			
	Intercept *b* (*SE*)	*P*-values		Slope *b* (*SE*)	*P*-values	Intercept *b* (*SE*)	*P*-values	Slope *b* (*SE*)	*P*-values	Intercept *b* (*SE*)	*P*-values	Slope *b* (*SE*)	*P*-values
Gender	**-0.17 (0.07)**	**0.010**		-0.01 (0.10)	0.911	-0.05 (0.06)	0.420	-0.05 (0.10)	0.611	-0.01 (0.08)	0.895	-0.07 (0.11)	0.549
Parental education level	0.06 (0.07)	0.355		0.10 (0.10)	0.310	0.11 (0.06)	0.078	0.09 (0.10)	0.380	0.04 (0.07)	0.578	0.12 (0.11)	0.253
Family monthly income (T0)	**0.18 (0.07)**	**0.006**		-0.04 (0.10)	0.682	**0.18 (0.06)**	**0.003**	-0.16 (0.10)	0.118	0.06 (0.07)	0.449	-0.06 (0.11)	0.596
BMI (T0)	-0.08 (0.06)	0.172		-0.01 (0.09)	0.928	-0.05 (0.06)	0.383	-0.04 (0.09)	0.653	-0.04 (0.07)	0.577	-0.05 (0.10)	0.619
FSC (T0 → T1)	0.09 (0.07)	0.164		0.01 (0.10)	0.912	0.01 (0.06)	0.821	0.02 (0.10)	0.877	0.04 (0.07)	0.563	-0.03 (0.11)	0.754
PSC (T0 → T1)	**-0.32 (0.06)**	** < 0.001**		**0.45 (0.12)**	** < 0.001**	**-0.31 (0.06)**	** < 0.001**	**0.50 (0.14)**	** < 0.001**	**-0.28 (0.07)**	** < 0.001**	**0.46 (0.14)**	** < 0.001**
χ2/df	28.67/7					20.21/7				21.30/7			
RMSEA	0.09					0.07				0.07			
SRMR	0.03					0.03				0.02			
CFI	0.93					0.95				0.93			

*FSC* Family support change, *PSC* Peer support change, T0, T1 Time 0 (the second year of high school) and 1 (the first year of college), respectively; gender coded as 0 = male and 1 = female. The Bonferroni adjusted significance level was set to 0.017 (0.05/3). Bonferroni-corrected significance level is boldface

When considering the effects of social support change on the trajectory of overall physical activity, the model showed acceptable model fit (χ^2^ (7) = 28.67, CFI = 0.93, RMSEA = 0.09, SRMR = 0.03). As shown in Table 2, peer support change was associated with intercept (*b* = -0.32, *p* < 0.001) and slope (*b* = 0.45, *p* < 0.001) for the trajectory of overall physical activity, indicating that students with a lower initial level at T0 (relative to T1) but a greater increase in peer support over time experienced lower initial levels of physical activity and a greater increase in physical activity across the transition from late adolescence to young adulthood. However, the effect of family support change on the trajectory of overall physical activity was insignificant.

When considering the effects of social support change on the trajectory of physical activity duration, the model showed an acceptable model fit (χ^2^ (7) = 20.21, CFI = 0.95, RMSEA = 0.07, SRMR = 0.03). As shown in Table 2, the association between family support change and the trajectory of duration was not significant; while peer support change was associated with intercept (*b* = -0.31, *p* < 0.001) and slope (*b* = 0.50, *p* < 0.001) for the trajectory of duration, indicating that students with a lower initial level at T0 (relative to T1) but a greater increase in peer support over time experienced lower initial levels but a greater increase in duration of physical activity across the transition from late adolescence to young adulthood.

When considering the effects of social support change on the trajectory of physical activity frequency, the model showed good model fit (χ^2^ (7) = 21.30, CFI = 0.93, RMSEA = 0.07, SRMR = 0.02). As shown in Table 2, the association between family support change and the trajectory of frequency was not significant; while peer support change was associated with intercept (*b* = -0.28, *p* < 0.001) and slope (*b* = 0.46, *p* < 0.001) for the trajectory of frequency, indicating that students with a lower initial level at T0 (relative to T1) but a greater increase in peer support over time experienced lower initial levels and a slower decline in frequency of physical activity across the transition from late adolescence to young adulthood.

### Sensitivity analyses

The study repeated the analyses for the *N* = 451 sample in a data set in which missing values were imputed using Maximum Likelihood Estimation [46, 47] and sociodemographic covariates (i.e., gender, parental education level, family monthly income, and BMI) were treated as control variables to account for independent variables and dependent variables. The results from sensitivity analyses were similar to those from the primary analyses (Table S1).

## Discussion

The study unpacked trajectories of physical activity (i.e., overall physical activity, duration, and frequency) during the transition from late adolescence to young adulthood, and evaluated the effects of social support (i.e., family and peer support) change on trajectories of physical activity. The latent growth models revealed an increase in overall physical activity and duration but a decrease in frequency during the transition from late adolescence (i.e., the second year of high school) to young adulthood (i.e., the third year of college). Further, family support change did not contribute to trajectories of physical activity, while peer support change significantly predicted the trajectory of overall physical activity, duration, and frequency. The current study demonstrated that there were differences in the change of duration and frequency of physical activity during the transition from adolescence to young adulthood and highlighted the importance of peer support over family support during the transition period to provide a better understanding of the predictors linked with physical activity trajectories.

### Trajectories of physical activity

The transition from late adolescence to young adulthood is an important time to develop unhealthy habits (e.g., inactive physical activity) as a result of psychosocial changes [10, 11]. However, inconsistent with previous studies, where a decrease in overall physical activity was observed during the transition from adolescence into young adulthood [48, 49], the current study observed an increase in overall physical activity over time. One explanation may be due to the decrease in pressure from the most competitive entrance exam in China—Gaokao, college students have more disposable time and money to engage in physical activity. Of note, the present study observed that different indicators (e.g., duration, frequency) of physical activity changed with different trajectories, such that duration showed a rising trend and frequency tended to decrease. The present study contributes to burgeoning literature examining the trajectory of physical activity by clarifying how the duration and frequency of physical activity change. These findings are inconsistent with a previous study conducted in Norway reporting a declining trend both for duration and frequency of physical activity during the period [50], and two recent systematic reviews indicating a decline in physical activity duration from adolescence to early adulthood [5, 51]. One of the explanations for the inconsistency may be related to the demographic characteristics of the samples and cultural contexts. Participants of the present study consisted of Chinese college students, whereas prior findings relied on both college students from other countries and people in the workforce [50]. A systematic review performed an analysis to consider the destination of those leaving high school and found that individuals continuing to college experienced a smaller decrease in physical activity compared with those not continuing in education, which means that different environments in which individuals go after leaving high school may lead to differences in physical activity [51]. For example, people in the workforce tend to report less time devoted to physical activity than those entering college [52]. This suggests that entering college may protect students against declines in physical activity duration. College may provide more physical activity resources and equipment as well as a social framework for participating in sport and physical activity that is more likely to be continued after leaving high school. Further, after completing the most competitive entrance exam in China—Gaokao, Chinese college students have more time to devote to other things (e.g., physical activity) because of the decrease in academic stress [53, 54]. Even if the frequency of physical activity decreases, college students’ duration of bouts of physical activity may be longer (relative to high school students) because of the absence of Gaokao pressure. In contrast, the decreasing trajectory of frequency occurred during the transition from late adolescence to young adulthood, in which the need for interpersonal relatedness is increasing and more opportunity for social contact is sought [55]. Thus, college students may exercise less frequently to free up more time for meeting other needs.

### Effects of social support change on trajectories of physical activity

The current study observed that peer support change predicted the intercept and slope of physical activity (including overall physical activity, duration, and frequency), such that students with a lower initial level at T0 (relative to T1) but a greater increase in peer support over time experienced lower initial levels but a greater increase in physical activity across the transition from late adolescence (i.e., high school) to young adulthood (i.e., college). This result was in line with prior empirical studies [37], indicating that individuals who experience higher levels of social support would increase their likelihood of engaging in physical activity. Due to the changes in social roles, academic and social environments, peer support change may occur during the transition period from high school to college, in which sufficient social resources can help young adults navigate important milestones [56]. Prior studies supported the notion that first-year college students with higher peer support for physical activity were more likely to report greater health beliefs, self-efficacy for coping with barriers, and sports-related resources [57, 58], which contributed to the higher levels of physical activity. Of note, the different effects of family support change and peer support change on trajectories of physical activity were observed in the present study, such that family support change did not significantly predict trajectories of physical activity, suggesting that peer support may be more important than parent support for those experiencing a transition from high school to college. These findings are similar to a previous study, reporting that parent support was important but less influential with age while the influence of peer support was increasing [18]. The difference may be related to development characteristics during the transition from late adolescence to young adulthood, in which individuals spend increasingly less time with family and more time with peers, and begin to establish greater independence from their parents, as well as their peers become an important social group [58]. Thus, trajectories of physical activity were more susceptible to the change in peer support (relative to family support) during the transition period. Taken together, the results from the current study extend the current literature by informing different effects of family and peer support change on trajectories of physical activity and highlighting the importance of understanding the associations between fluctuations in family support and peer support and physical activity from a dynamic perspective.

### Limitations and future directions

Several limitations should be noted when interpreting the findings. First, all data were collected with self-reports, which could yield a threat to internal validity.

Objective or direct measures (e.g., accelerometers, pedometers, and heart rate monitors) of physical activity may be useful in future research to increase precision and accuracy by reducing the effects of recall and response bias and overly positive self-presentation [59]. Second, the assessment of physical activity in the second year of high school was retrospective, which cannot preclude the existence of recall bias. Third, the samples in the study were recruited solely from a single university in China, which may further impact the generalizability. It would be informative for future research to use bigger samples with more regionally, socioeconomically, and culturally diverse backgrounds to examine the effects of social support change on the physical activity trajectory. Fourth, while researchers conceptualize perceived social support for physical activity as a multidimensional construct (e.g., tangible support, encouragement, involvement) [60], the present study only assessed social support as a unidimensional construct, which masked nuanced but potentially distinctive effects in different types of social support, such that the effect sizes in the associations between parental encouragement and child physical activity were bigger than those observed in the associations between instrumental support and child physical activity [61]. It would be informative for future work to examine the types of family and peer support change and trajectories of physical activity. Meanwhile, social support change was assessed with a single-item measure, which cannot cover sufficient territory of the target construct and cannot estimate scale reliability (e.g., internal consistency). Although single-item measures have been used to assess social support in prior studies [40, 41], it is important to replicate the present findings with better-validated multi-item measures.

## Conclusion

Extant research has suggested that higher levels of social support are associated with more physical activity among adolescents and early adults. Nevertheless, most of these findings were based on cross-sectional data; how physical activity changes during the transition from late adolescence to young adulthood and the predictive roles of social support change remained unclear. The current study contributes uniquely to the literature on physical activity by revealing an increase in overall physical activity and duration but a decrease in frequency during the transition from late adolescence to young adulthood, and provides a better understanding of associations between social support change and trajectories of physical activity by unpacking the significant effects of peer support change and the insignificant effects of family support change on trajectories of physical activity. The findings could inform practitioners who aim to increase the physical activity of students during the transition from late adolescence to young adulthood, such that they may find it especially beneficial to provide more peer support and resources for those with low levels of physical activity.

### Supplementary Information

**Additional file 1:** **Table S1.** Effects of family support change and peer support change on trajectories of physical activity (*N *= 451).

## Data Availability

The data and code from the current study are available from an online repository (https://www.mediafire.com/folder/84r6smq8zjrf5/Data).
