# Supplementary material for: The associations between social support change and physical activity trajectory from late adolescence to young adulthood
Source: BMC Public Health. 2023 Aug 7;23:1496. doi: 10.1186/s12889-023-16422-z (PMC10405443; doi:10.1186/s12889-023-16422-z)
Supplement: Supplementary file 1 — Additional file 1: Table S1. Effects of family support change and peer support change on trajectories of physical activity (N = 451). [file 12889_2023_16422_MOESM1_ESM.docx]

**Table S1** Effects of family support change and peer support change on trajectories of physical activity (*N* = 451)

|  | Overall physical activity | | | |  | Duration | | | |  | Frequency | | | |
| --- | --- | --- | --- | --- | --- | --- | --- | --- | --- | --- | --- | --- | --- | --- |
|  | Intercept  *b* (*SE*) | *P*-values | Slope  *b* (*SE*) | *P*-values |  | Intercept  *b* (*SE*) | *P*-values | Slope  *b* (*SE*) | *P*-values | | Intercept  *b* (*SE*) | *P*-values | Slope  *b* (*SE*) | *P*-values |
| Gender | **-0.19 (0.06)** | **0.003** | 0.03 (0.10) | 0.782 |  | -0.05 (0.06) | 0.422 | -0.06 (0.10) | 0.539 |  | -0.01 (0.07) | 0.882 | -0.02 (0.10) | 0.859 |
| Parental education level | **0.16 (0.06)** | **0.007** | 0.04 (0.09) | 0.654 |  | **0.20 (0.06)** | **<0.001** | 0.02 (0.09) | 0.836 |  | 0.08 (0.07) | 0.209 | 0.06 (0.10) | 0.531 |
| Family monthly income (T0) | 0.04 (0.06) | 0.550 | -0.05 (0.10) | 0.587 |  | 0.07 (0.06) | 0.200 | -0.16 (0.10) | 0.126 |  | -0.02 (0.07) | 0.785 | -0.07 (0.11) | 0.512 |
| BMI (T0) | -0.01 (0.06) | 0.879 | 0.07 (0.10) | 0.472 |  | -0.01 (0.06) | 0.806 | 0.01 (0.09) | 0.945 |  | 0.02 (0.07) | 0.799 | 0.04 (0.10) | 0.725 |
| FSC (T0→T1) | -0.14 (0.06) | 0.024 | 0.25 (0.11) | 0.022 |  | -0.09 (0.06) | 0.113 | 0.17 (0.10) | 0.095 |  | -0.04 (0.07) | 0.569 | 0.09 (0.10) | 0.400 |
| PSC (T0→T1) | **-0.24 (0.06)** | **<0.001** | **0.40 (0.13)** | **0.002** |  | **-0.27 (0.06)** | **<0.001** | **0.46 (0.14)** | **0.001** |  | **-0.25 (0.07)** | **<0.001** | **0.42 (0.14)** | **<0.001** |
| χ2/df | 22.58/7 | | | |  | 20.37/7 | | | |  | 14.41/7 | | | |
| RMSEA | 0.07 | | | |  | 0.07 | | | |  | 0.05 | | | |
| SRMR | 0.03 | | | |  | 0.03 | | | |  | 0.02 | | | |
| CFI | 0.95 | | | |  | 0.95 | | | |  | 0.96 | | | |

*Note.* FSC= Family support change; PSC= Peer support change; T0, T1 = Time 0 (the second year of high school) and 1 (the first year of college), respectively; gender coded as 0 = male and 1 = female. The Bonferroni adjusted significance level was set to 0.017 (0.05/3). Sociodemographic covariates (i.e., Gender, Parental education level, Family monthly income, and BMI) as control variables to account for independent variables and dependent variables. Bonferroni-corrected significance level is boldface.
